# Supplementary material for: Coverage and timeliness of vaccination and the validity of routine estimates: Insights from a vaccine registry in Kenya
Source: Vaccine. 2018 Dec 18;36(52):7965–74. doi: 10.1016/j.vaccine.2018.11.005 (PMC6288063; doi:10.1016/j.vaccine.2018.11.005)
Supplement: Supplementary Data 2 [file mmc2.docx]

**Table S1. Vaccination coverage and timeliness of each vaccine in residents of the Kilifi Health and Demographic Surveillance System (KHDSS) aged 12-23 months by birth cohort and survey population aged 12-23 months (other vaccine doses)**

| **Vaccine** | **Cohort** | **Coverage birth cohort**  **(95% CI)** | **Median age (weeks)** | **Timely vaccination (%)*** | **AUC****  **(%)** | **Year** | **Coverage 12-23 months**  **(95% CI)** | **Median age (weeks)** | **Timely vaccination (%)*** |
| --- | --- | --- | --- | --- | --- | --- | --- | --- | --- |
| **OPV1** |  |  |  |  |  |  |  |  |  |
|  | 2010-11 | 2010-11 | 89.8 (89.1-90.4) | 6.4 | 84.4 | 87.8 | 2012 | 92.0 (91.4-92.6) | 6.4 |
|  | 2011-12 | 2011-12 | 90.0 (89.3-90.6) | 6.3 | 84.9 | 88.2 | 2013 | 92.3 (91.7-92.9) | 6.3 |
|  | 2012-13 | 2012-13 | 90.4 (89.7-91.0) | 6.4 | 82.3 | 88.1 | 2014 | 93.8 (93.2-94.4) | 6.4 |
|  | 2013-14 | 2013-14 | 92.8 (92.2-93.4) | 6.3 | 88.3 | 91.2 | 2015 | 96.0 (95.5-96.4) | 6.3 |
|  | 2014-15 | 2014-15 | 94.1 (93.6-94.6) | 6.3 | 81.6 | 91.1 | 2016 | 95.7 (95.2-96.1) | 6.3 |
|  | 2015-16 | 2015-16 | 88.5 (87.7-892) | 6.4 | 70.2 | 84.6 | 2017 | 89.9 (89.1-90.6) | 6.4 |
|  |  |  |  |  |  |  |  |  |  |
| **OPV2** |  |  |  |  |  |  |  |  |  |
|  | 2010-11 | 2010-11 | 89.2 (88.5-89.3) | 11.0 | 78.5 | 85.5 | 2012 | 91.3 (90.7-91.9) | 10.8 |
|  | 2011-12 | 2011-12 | 89.0 (88.3-89.7) | 10.8 | 79.8 | 85.7 | 2013 | 91.3 (90.6-92.0) | 10.8 |
|  | 2012-13 | 2012-13 | 89.3 (88.6-90.0) | 11.0 | 74.4 | 84.9 | 2014 | 92.7 (92.1-93.3) | 11.0 |
|  | 2013-14 | 2013-14 | 92.5(91.9-93.0) | 10.7 | 84.0 | 89.3 | 2015 | 95.5 (95.0-96.0) | 10.7 |
|  | 2014-15 | 2014-15 | 93.2 (92.6-93.8) | 11.0 | 70.7 | 87.1 | 2016 | 94.5 (93.9-95.0) | 11.0 |
|  | 2015-16 | 2015-16 | 87.6 (86.9-88.4) | 11.5 | 58.0 | 80.2 | 2017 | 88.7 (87.9-89.5) | 11.4 |
|  |  |  |  |  |  |  |  |  |  |
| **Penta 1** |  |  |  |  |  |  |  |  |  |
|  | 2010-11 | 2010-11 | 89.6 (88.9-90.2) | 7.0 | 67.1 | 84.2 | 2012 | 91.6 (91.0-92.3) | 6.8 |
|  | 2011-12 | 2011-12 | 89.9 (89.2-90.6) | 6.4 | 81.4 | 87.6 | 2013 | 92.2 (91.6-94.4) | 6.4 |
|  | 2012-13 | 2012-13 | 90.4 (89.8-91.1) | 6.4 | 83.2 | 88.3 | 2014 | 93.8 (93.3-94.4) | 6.3 |
|  | 2013-14 | 2013-14 | 92.9 (92.3-93.4) | 6.3 | 88.8 | 91.3 | 2015 | 96.1(95.6-96.5) | 6.3 |
|  | 2014-15 | 2014-15 | 94.2 (93.6-94.7) | 6.3 | 91.2 | 92.9 | 2016 | 95.8 (95.3-96.3) | 6.1 |
|  | 2015-16 | 2015-16 | 88.7 (87.9-89.4) | 6.1 | 86.6 | 87.6 | 2017 | 90.3 (89.5-91.0) | 6.1 |
| **Penta2** |  |  |  |  |  |  |  |  |  |
|  | 2010-11 | 88.0 (87.3-88.7) | 14.0 | 50.2 | 71.0 | 2012 | 90.0 (89.3-90.7) | 14.0 | 51.4 |
|  | 2011-12 | 89.0 (87.3-88.7) | 11.0 | 74.7 | 77.5 | 2013 | 91.3 (90.6-91.9) | 10.8 | 76.7 |
|  | 2012-13 | 89.4 (88.3-89.7) | 11.0 | 76.0 | 77.9 | 2014 | 92.9 (92.2-93.5) | 10.8 | 79.5 |
|  | 2013-14 | 92.5 (91.9-93.0) | 10.7 | 84.6 | 81.8 | 2015 | 95.5 (95.0-96.0) | 10.7 | 87.7 |
|  | 2014-15 | 93.8 (93.3-94.4) | 10.5 | 87.9 | 83.5 | 2016 | 95.3 (94.8-95.8) | 10.5 | 89.6 |
|  | 2015-16 | 88.0 (87.2-88.7) | 10.5 | 84.0 | 78.6 | 2017 | 89.5 (88.8-90.3) | 10.5 | 85.7 |
|  |  |  |  |  |  |  |  |  |  |
| **PCV1** |  |  |  |  |  |  |  |  |  |
|  | 2010-11 | 88.6 (87.9-89.3) | 10.1 | 49.8 | 76.2 | 2012 | 90.5 (89.8-91.1) | 9.8 | 51.3 |
|  | 2011-12 | 89.8 (89.1-90.4) | 6.3 | 85.4 | 88.1 | 2013 | 92.1 (91.5-92.7) | 6.3 | 87.7 |
|  | 2012-13 | 90.0 (89.3-90.6) | 6.4 | 82.9 | 87.8 | 2014 | 93.5 (92.9-94.1) | 6.3 | 86.5 |
|  | 2013-14 | 92.8 (92.2-93.3) | 6.3 | 88.5 | 91.2 | 2015 | 96.0 (95.5-96.4) | 6.3 | 91.9 |
|  | 2014-15 | 94.1 (93.6-94.7) | 6.3 | 91.1 | 92.9 | 2016 | 95.8 (95.3-96.3) | 6.3 | 92.9 |
|  | 2015-16 | 88.6 (87.9-89.3) | 6.1 | 86.3 | 87.6 | 2017 | 90.2 (89.5-90.9) | 6.1 | 88.0 |
|  |  |  |  |  |  |  |  |  |  |
| **PCV2** |  |  |  |  |  |  |  |  |  |
|  | 2010-11 | 87.1 (86.3-87.8) | 15.0 | 45.8 | 65.7 | 2012 | 88.9 (88.1-89.5) | 14.5 | 47.3 |
|  | 2011-12 | 89.0 (88.2-89.7) | 10.8 | 81.0 | 78.5 | 2013 | 91.3 (90.6-91.9) | 10.8 | 83.2 |
|  | 2012-13 | 89.1 (88.4-89.7) | 11.0 | 75.6 | 77.7 | 2014 | 92.6 (91.9-93.1) | 10.8 | 79.2 |
|  | 2013-14 | 92.4 (91.8-93.0) | 10.7 | 84.2 | 81.7 | 2015 | 95.5 (95.0-96.0) | 10.7 | 87.3 |
|  | 2014-15 | 93.8 (93.3-94.3) | 10.5 | 87.7 | 83.4 | 2016 | 95.4 (94.5-95.5) | 10.5 | 89.5 |
|  | 2015-16 | 87.9 (87.2-88.7) | 10.5 | 83.2 | 78.5 | 2017 | 89.5 (88.8-90.3) | 10.5 | 85.0 |

BCG Bacille Calmette-Guérin vaccine (BCG), OPV3 Oral Polio Vaccine 3^rd^ dose, Pentavalent Vaccine 3^rd^ dose (Diphtheria, Pertussis, Tetanus, *Haemophilus influenzae* b and Hepatitis B combination vaccine), PCV 3 Pneumococcal Conjugate Vaccine 3^rd^ dose, MCV1 Measles-Containing Vaccine 1^st^ dose.

* Proportion of vaccinated children who received their vaccines within 4 weeks of become age-eligible for vaccination

** AUC % Area Under the Curve (see figure 1B)

**Table S2. Predictors of vaccination and timeliness among children in the KHDSS by birth cohort**

|  | **Univariate analyses** | | | |  | **Multivariable analyses** | | | |
| --- | --- | --- | --- | --- | --- | --- | --- | --- | --- |
|  | **PCV3** | | **OPV3** | |  | **PCV3** | | **OPV3** | |
| **Risk factors** | **HR*** | **95% CI** | **HR** | **95% CI** |  | **HR** | **95% CI** | **HR** | **95% CI** |
| Time trend (years) | 1.11 | 1.12-1.13 | 0.95 | 0.94-0.96 |  | 1.13 | 1.12-1.13 | 0.95 | 0.94-0.96 |
|  |  |  |  |  |  |  |  |  |  |
| Male sex | 0.99 | 0.98-1.01 | 0.99 | 0.97-1.01 |  | 0.99 | 0.97-1.01 | 0.98 | 0.96-1.00 |
|  |  |  |  |  |  |  |  |  |  |
| Maternal age (years) |  |  |  |  |  |  |  |  |  |
| <25 | - |  | - |  |  | - |  | - |  |
| 25-34 | 1.02 | 1.00-1.04 | 1.03 | 1.01-1.05 |  | 0.98 | 0.93-0.98 | 0.98 | 0.96-1.01 |
| ≥35 | 1.01 | 0.98-1.04 | 1.00 | 0.98-1.04 |  | 0.96 | 0.92-0.99 | 0.95 | 0.92-0.98 |
|  |  |  |  |  |  |  |  |  |  |
| Place of birth |  |  |  |  |  |  |  |  |  |
| Home | - |  | - |  |  | - |  | - |  |
| Health facility | 1.36 | 1.33-1.38 | 1.13 | 1.11-1.16 |  | 1.26 | 1.24-1.29 | 1.25 | 1.22-1.27 |
|  |  |  |  |  |  |  |  |  |  |
| Distance from clinic |  |  |  |  |  |  |  |  |  |
| <3 km | - |  | - |  |  | - |  | - |  |
| ≥3 km | 0.98 | 0.96-0.99 | 0.96 | 0.94-0.98 |  | 0.99 | 0.97-0.98 | 0.96 | 0.96-1.00 |
|  |  |  |  |  |  |  |  |  |  |
| Vaccine stock out | 0.66 | 0.57-0.77 | 0.54 | 0.52-0.57 |  | 0.58 | 0.49-0.68 | 0.56 | 0.53-0.58 |
|  |  |  |  |  |  |  |  |  |  |
| Birth order |  |  |  |  |  |  |  |  |  |
| <2 | - |  | - |  |  | - |  | - |  |
| 2-5 | 1.03 | 1.01-1.06 | 1.07 | 1.06-1.10 |  | 1.12 | 1.09-1.15 | 1.12 | 1.09-1.15 |
| >5 | 1.00 | 0.97-1.02 | 1.04 | 1.01-1.06 |  | 1.10 | 1.06-1.14 | 1.10 | 1.06-1.13 |

*HR, Hazard ratios indicate the increased ‘hazard’ of being vaccinated among each of the risk factor categories, compared to baseline

**Adjusted for all other variables-year of birth, sex, maternal age, place of birth, distance from clinic, stockout and birth order.
